# Association between Dietary Vitamin A Intake and the Risk of Glioma: Evidence from a Meta-analysis

**DOI:** 10.3390/nu7115438

**Published:** 2015-10-28

**Authors:** Wen Lv, Xian Zhong, Lingmin Xu, Weidong Han

**Affiliations:** 1 Department of Internal Neurology, Sir Run Run Shaw Hospital, College of Medicine, Zhejiang University, Hangzhou 310016, China; wenlv123@yeah.net; 2 Department of Medical Oncology, Hangzhou Binjiang Hospital, Hangzhou 310052, China; xianzhong123@yeah.net; 3 Department of Medical Oncology, Sir Run Run Shaw Hospital, College of Medicine, Zhejiang University, Hangzhou 310016, China; lingminxu123@yeah.net

**Keywords:** vitamin A, glioma, meta-analysis

## Abstract

The results from epidemiological studies between dietary vitamin A intake and glioma risk is not consistent. Thus, a meta-analysis was conducted to confirm the exact relationship between them. PubMed and Web of Knowledge were used to search the relevant articles up to May 2015. Pooled relative risk (RR) with 95% confidence interval (CI)was calculated using random-effect model. Egger’s test was used to assess the small-study effect. At the end, seven articles with eight case-control studies involving 1841 glioma cases and 4123 participants were included. Our study indicated that highest category of dietary vitamin A intake was significantly associated with reduced risk of glioma (RR = 0.80, 95% CI = 0.62–0.98, *p* = 0.014, *I*^2^ = 54.9%). Egger’s test did not find any publication bias. In conclusion, our study indicated that higher category of dietary vitamin A intake could reduce the glioma risk. However, we could not do a dose-response analysis for vitamin A intake with glioma risk due to the limited data in each reported individual article. Due to this limitation, further studies with detailed dose, cases and person-years for each category is wanted to assess this dose-response association.

## 1. Introduction

Approximately 70% of adult brain tumors are glioma, and it is the most common primary brain tumor that occurs most frequently in brain among adults [1,2]. Two recent meta-analyses had been published to confirm the associations between vitamin C [3] and vitamin E [4] and glioma risk. The results concluded that higher category of dietary vitamin C intake could reduce the glioma risk and dietary vitamin E intake could not affect the glioma risk. Dietary antioxidants, including vitamin A and vitamin C intake, have been shown in laboratory studies to enhance growth restriction of cancer cells in general [5] and glioma cells in particular [6,7]. Vitamin A, vitamin C, and vitamin E are all antioxidant nutrients. And they may influence the process since their being free radical scavengers, indicating that they could play an important role in glioma prevention. To date, many epidemiological studies were conducted to explore the relationship between vitamin A intake and glioma risk. Two studies obtain an inverse association between dietary vitamin A intake and glioma risk [8,9], while five studies found a negative association between them [10,11,12,13,14]. Furthermore, Giles *et al.* found an increased risk of glioma in males with higher category of dietary vitamin A intake [11]. Considering the results are not consistent, we therefore conduct this comprehensive meta-analysis to assess the association between them.

## 2. Methods

### 2.1. Search Strategy

The relevant articles were searched using the databases of PubMed [15] and Web of Knowledge [16] up to May 2015. We also reviewed the computer retrieved studies for reference lists by hand-searching. We used the following search terms: “glioma” or “brain cancer” combined with “vitamin A” or “antioxidants” or “lifestyle” or “diet”. Two investigators (Wen Lv and Xian Zhong) searched the relevant articles and independently reviewed all retrieved studies.

### 2.2. Inclusion Criteria

The following inclusion criteria were used: (1) the studies were using a prospective design or a case-control design; (2) the exposure of interest was dietary vitamin A intake; (3) the ending outcome was glioma; (4) odds ratio (OR) or relative risks (RR) and their 95% confidence intervals (CI) were available for highest category of dietary vitamin A *vs.* lowest category of dietary vitamin A intake.

### 2.3. Data Extraction

Two researchers (Wen Lv and Xian Zhong) extracted the following information from the included study independently: the last name of the first author’s, publication years, geographic locations of the study, study design, sample source, the age for cases and participants, the number of cases and person-years, and RR (95% CI) for the highest category of dietary vitamin A intake *versus* lowest category of vitamin A intake. The multivariable adjustment RR (95% CI) was used from each reported study if possible.

### 2.4. Statistical Analysis

The multivariate adjusted RR with 95% CI for highest category of vitamin A *vs.* lowest category for the glioma risk was pooled using a random-effects model [17]. The *I*^2^ of Higgins and Thompson [18] was used to assess the between-study heterogeneity. *I*^2^ described the proportion of total variation attributable to between-study heterogeneity, and *I*^2^ values of 0, 25%, 50% and 75% represent no, low, moderate, and high heterogeneity, respectively [19]. Meta-regression and subgroup analyses (geographic locations and study design) were performed to explore the potential between-study heterogeneity [20]. Egger’s test was used to evaluate the small-study effect [21]. A sensitivity analysis was conducted to describe if the pooled RR lies outside the 95% CI when removal of one individual studies at a time [22]. If so, then the one study was considered to be an impact on the overall result. We used STATA software, version 10.0 to analyze all the data (StataCorp LP, College Station, TX, USA). Two-tailed *p* ≤ 0.05 was accepted as statistically significant.

## 3. Results

### 3.1. Characteristics of Included Studies

**Figure 1:**
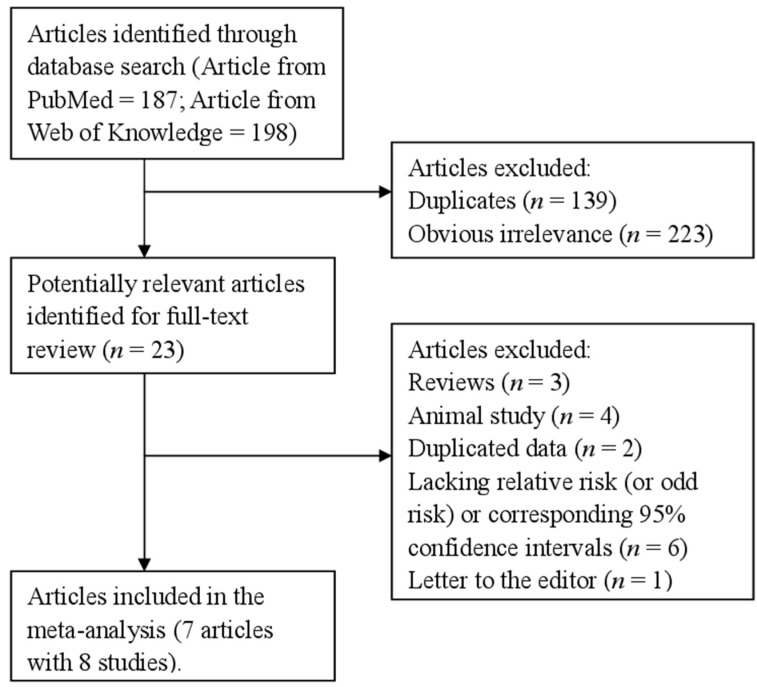
The detailed steps of our literature search.

We identified 385 articles from all the databases, and twenty-three articles were reviewed in full after reviewing the title/abstract. Sixteen studies were further excluded from this analysis for various reasons showed in Figure 1. Therefore, seven articles [8,9,10,11,12,13,14] with 8 case-control studies comprising 1841 glioma cases and 4123 participants were used in this study. Table 1 showed the characteristics of the included studies. Five studies were conducted in United States, two studies in Australia, and one study in China.

### 3.2. Vitamin A and Glioma

**Figure 2:**
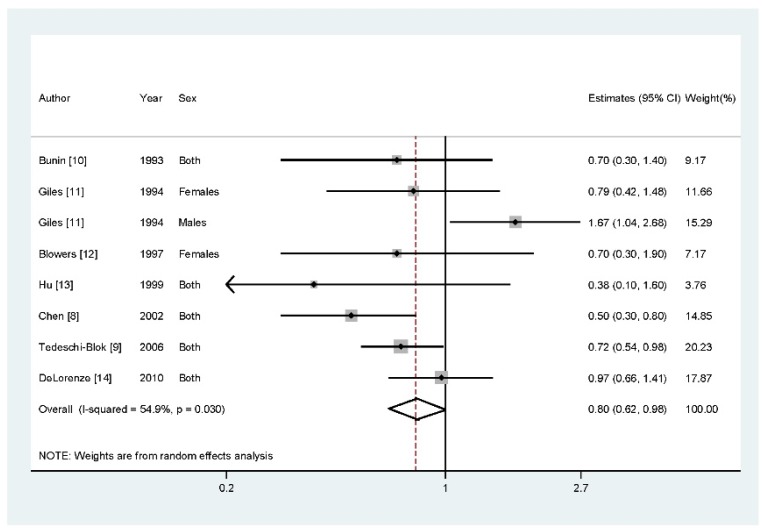
The forest plot between highest *versus* lowest categories of vitamin A intake and glioma risk.

Data from seven articles with eight studies involving 1841 glioma cases were included in this study. Two studies reported that higher vitamin A intake could reduce the risk of glioma, while five studies found a negative association between them. However, there is one study found an increased risk of glioma with higher category of vitamin A intake. Results from our study suggested that highest category of dietary vitamin A intake *versus* lowest category could reduce the glioma risk (RR = 0.80, 95% CI = 0.62–0.98, *p* = 0.014, *I*^2^ = 54.9%) (Figure 2).

### 3.3. Meta-Regression

Moderate of heterogeneity (*I*^2^ = 54.9%, *p*_heterogeneity_ = 0.030) was detected in our results. We then used meta-regression with the covariates of publication years, geographic locations where the study conducted, cases and source of controls to explore the potential heterogeneity. However, no significant finding was found in the above-mentioned analysis (the detailed results were shown in Supplement Table A1).

### 3.4. Influence Analysis and Publication Bias

**Figure 3:**
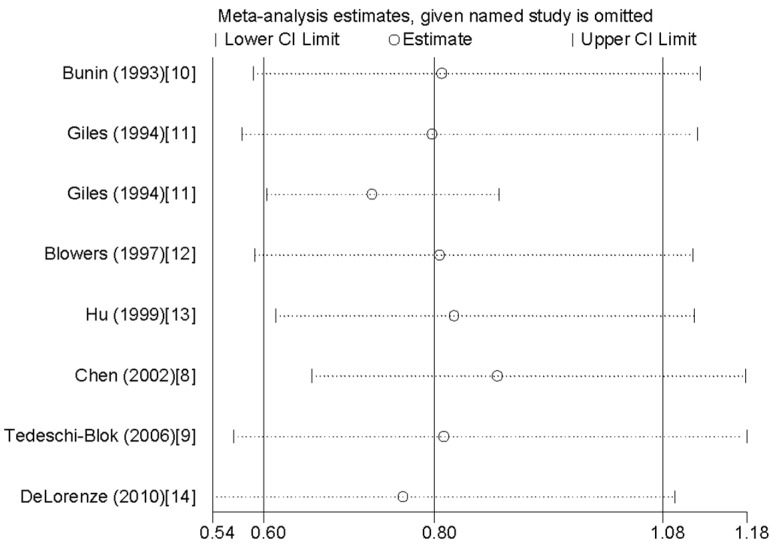
Analysis of influence of individual study on the association between vitamin A intake and glioma risk. Open circle indicates the pooled relative risk, given named study is omitted. Horizontal lines represent the 95% CI.

Influence analysis showed that the pooled RR did not lie out of the 95% CI when we removed one individual study at a time (Figure 3). There is no publication bias found by Egger’s regression asymmetry test (*p* = 0.564).

In stratified analysis by geographic locations, highest category of dietary vitamin A intake could reduce glioma risk among American populations (RR = 0.73, 95% CI = 0.59–0.91, *p* < 0.001), but not in the other countries (summary RR = 0.95, 95% CI = 0.45–2.01). Seven of the included studies were population-based case-control (PBCC) studies, and only one study was hospital-based case-control (HBCC) study. We then combined the data for PBCC studies, but not for HBCC studies. And the result was only significant in PBCC studies (summary RR = 0.83, 95% CI = 0.68–0.98, *p* = 0.011).

**Table 1:** Characteristics of studies on vitamin A intake and glioma risk.

First Author, Year	Country	Study Design	Cases, Age (year)	Comparison Groups	Dietary Assessment	RR (95% CI) for Highest *versus* Lowest Category	Adjustment or Matched for
Bunin *et al.* 1994 [10]	United States	Case-control (PCC)	155, <6	Quartile 4 *vs.* Quartile 1	FFQ	0.70 (0.30–1.40)	Adjusted for income level.
Giles *et al.* 1994 [11]	Australia	Case-control (PCC)	416, 20–70	Tertile 3 *vs.* Tertile1	FFQ	Females: 0.79 (0.42–1.48); Males: 1.67 (1.04–2.68)	Adjusted for alcohol and tobacco.
Blowers *et al.* 1997 [12]	United States	Case-control (PCC)	94, 25–74	Quartile 4 *vs.* Quartile 1	FFQ	0.70 (0.30–1.90)	Matched the patient on age (within five years) and race (Black or White).
Hu *et al.* 1999 [13]	China	Case-control (HCC)	73, 20–74	Quartile 4 *vs.* Quartile 1	FFQ	0.38 (0.10–1.60)	Matched to each case by sex, age within five-year intervals and area of residence (same or adjacent city and country).
Chen *et al.* 2002 [8]	United States	Case-control (PCC)	236, ≥21	Quartile 4 (mean = 5078) *vs.* Quartile 1 (mean = 1269.5)	FFQ	0.50 (0.30–0.80)	Adjusting for age, age squared, gender, total energy intake, respondent type, education level, family history, and farming experience.
Tedeschi-Blok *et al.* 2006 [9]	United States	Case-control (PCC)	802, ≥20	Quartile 4 (mean = 5512) *vs.* Quartile 1 (mean = 1378)	FFQ	0.72 (0.54–0.98)	Adjusted for age, gender, ethnicity, SES, total calories, and supplement use
DeLorenze *et al.* 2010 [14]	United States	Case-control (PCC)	72, ≥20	Tertile 3 (mean = 3757.6) *vs.* Tertile 1 (mean = 257.7)	FFQ	0.97 (0.66–1.41)	Adjusted for reporting status, age at diagnosis, treatment, education, marital status, total calories, pack years, and age at alcoholic drink.

Abbreviations: RR: relative risk; CI: confidence intervals; PCC: population-based case–control study; HCC: hospital-based case–control study; FFQ: food-frequency questionnaire; SES: socio-economic status.

## 4. Discussion

Findings from this study indicated that higher category of vitamin A intake may prevent against of glioma risk. The association was also significant among American populations for vitamin A intake and glioma risk.

The mechanisms of vitamin A for preventing glioma risk have not been thoroughly investigated. Previous studies had suggested that antioxidants, such as vitamin A, vitamin C and vitamin E might prevent the risk of glioma. A recent meta-analysis had reported that higher category of dietary vegetables and fruits intake could prevent against the glioma [23]. Vegetables and fruit included antioxidants vitamins, beta-carotene, fiber, and folate, this may be a protective effects of glioma prevention [24]. Vitamin A is involved in cell growth, normal synthesis of DNA methylation, and protection against oxidative stress and DNA damage.

One paper had reported that heterogeneity in the meta-analysis is common [25], and exploring the potential sources is therefore an essential component of meta-analysis. In our pooled results, moderate of heterogeneity (*I*^2^ = 54.9%, *p*_heterogeneity_ = 0.030) was found. As we all know, the publication years, geographic location where the study conducted, and source of controls might arise the heterogeneity. We therefore performed meta-regression and subgroup analyses to explore the potential heterogeneity. As a result, we did not find the potential heterogeneity from the above analyses. Considering other genetic and environment variables may affect the heterogeneity, further studies with multivariate adjustment RR should assess this association.

Our meta-analysis had some advantages. To the best of our knowledge, we conducted the first comprehensive meta-analysis between dietary vitamin A intake and glioma risk based on highest level *versus* lowest level. Second, our meta-analysis included much more cases and participants; this may obtain a more reasonable result. Third, there is no significant publication bias tested by the Egger’ test. However, some limitations should be concerned in this study. First, the included studies were all case-control design. Case-control studies may suffer from recall bias and selection bias, but they are important methods in etiology research. Further studies original with prospective design and other design are wanted to confirm this result. Second, different measurement could affect the dietary intake. And the range of intake may be changed during the different measurement [26]. Third, we did not do the dose-response analysis due to the limited data reported in each individual study. Fourth, one study (Giles *et al.* 1994) [11] strongly suggested an association in the opposite way for a male study. However, we could not conduct the subgroup analysis by sex due to the limited data from each individual study, since only one study (Giles *et al.* 1994) [11] reported the association for males and females respectively and one study (Blowers *et al.* 1997) [12] reported the association for females only. Further studies should assess these associations. Fifth, different genetic aberrations are associated with susceptibility and onset of glioma. Evidences for genetic aberration leading to defects in the regulation of expression of enzymes that are involved in the biosynthesis of retinoic acid and its association with susceptibility to glioma exist [27,28]. But we could not conduct the subgroup analysis by gene and subtypes due to the limited data in each individual study. Further studies with detailed information should explore these associations. Finally, moderate between-study heterogeneity was found in our study, but the heterogeneity was not explained by subgroup analyses and meta-regression. However, other environment variables may affect this disease-effect unconformity.

## 5. Conclusions

In conclusions, findings from this study indicated that higher category of dietary vitamin A intake could reduce glioma risk. However, dose-response analysis was not performed between vitamin A intake and glioma risk due to the limited data reported in each individual article. Due to this limitation, further studies with detailed dose, cases, and participants (or person-years) for each category is wanted to assess this dose-response association.

## Appendix

**Table A1:** The detailed results for the covariates by univariate meta-regression.

Covariates	*p*-Value
Publication year	0.185
Geographic locations	0.294
Number of cases	0.386
Source of controls	0.815
